# Morphology of Thin Film Composite Membranes Explored by Small-Angle Neutron Scattering and Positron-Annihilation Lifetime Spectroscopy

**DOI:** 10.3390/membranes10030048

**Published:** 2020-03-18

**Authors:** Vitaliy Pipich, Marcel Dickmann, Henrich Frielinghaus, Roni Kasher, Christoph Hugenschmidt, Winfried Petry, Yoram Oren, Dietmar Schwahn

**Affiliations:** 1 Jülich Centre for Neutron Science JCNS-FRM II; Outstation at FRM II, Lichtenbergstr. 1, D-85747 Garching, Germany; v.pipich@fz-juelich.de (V.P.); h.frielinghaus@fz-juelich.de (H.F.); 2 Heinz Maier-Leibnitz Zentrum (MLZ), Technische Universität München, Lichtenbergstr. 1, D-85748 Garching, Germany; marcel.dickmann@frm2.tum.de (M.D.); christoph.hugenschmidt@frm2.tum.de (C.H.); winfried.petry@frm2.tum.de (W.P.); 3 Zuckerberg Institute for Water Research, Jacob Blaustein Institutes for Desert Research, Ben-Gurion University of the Negev, Sede Boqer Campus 84990, Israel; kasher@bgu.ac.il (R.K.); yoramo@bgu.ac.il (Y.O.)

**Keywords:** detection of the order of Å to micrometer large pores in RO membranes, fibers of nonwoven fabric support layer, chemistry and internal structures, positron-annihilation lifetime spectroscopy, small-angle neutron scattering using contrast variation

## Abstract

The morphology of thin film composite (TFC) membranes used in reverse osmosis (RO) and nanofiltration (NF) water treatment was explored with small-angle neutron scattering (SANS) and positron-annihilation lifetime spectroscopy (PALS). The combination of both methods allowed the characterization of the bulk porous structure from a few Å to µm in radius. PALS shows pores of ~4.5 Å average radius in a surface layer of about 4 μm thickness, which become ~40% smaller at the free surface of the membranes. This observation may correlate with the glass state of the involved polymer. Pores of similar size appear in SANS as closely packed pores of ~6 Å radius distributed with an average distance of ~30 Å. The main effort of SANS was the characterization of the morphology of the porous polysulfone support layer as well as the fibers of the nonwoven fabric layer. Contrast variation using the media H_2_O/D_2_O and supercritical CO_2_ and CD_4_ identified the polymers of the support layers as well as internal heterogeneities.

## 1. Introduction

**Figure 1:**
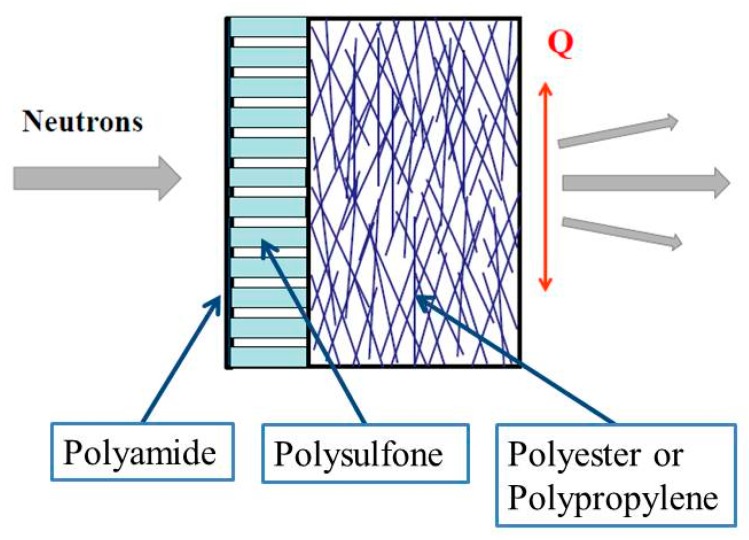
Design of thin film composite (TFC), reverse osmosis (RO), and nanofiltration (NF) membranes and pathway of the neutron beam through the membrane. The dimension of the membrane morphology such as pores is determined in direction of the scattering vector (**Q**). Thickness of the polyamide skin layer is in the range 0.1 to 0.3 µm, the porous and nonwoven fabric support layers about 40 μm and 100 to 300 μm, respectively.

Reverse osmosis (RO) and nanofiltration (NF) play a crucial role in the production of potable water from waste, brackish and seawater [1,2,3]. The main active components of RO desalination plants are thin film composite (TFC) membranes composed of three polymeric layers with a total thickness of ~140 to ~300 μm. Figure 1 shows a schematic description of TFC-RO/NF membranes. The layer facing the treated water, i.e., the feed is a polyamide (PA) film of thickness in the range 0.1 to 0.3 µm depending on membrane type deposited on top of a polyethersulfone (PES) or polysulfone (PSU) porous support layer of ~40 μm thickness. A second support layer is a nonwoven fabric made from polyester (PET) or polypropylene. 

In this paper, we analyze the morphology of several commercial RO and NF membranes from the perspectives of positron-annihilation lifetime spectroscopy (PALS) and small-angle neutron scattering (SANS). Both methods are non-invasive techniques, which usually do not require special sample treatment. PALS measures pores of a few Å radius in the membrane surface layer over a depth of about 3 µm thickness thereby exploring the attendance of micro pores in the whole polyamide skin layer as well as in the outer part of the polysulfone support layer. Thus, PALS delivers relevant structural information of the PA selective layer, which determines water permeability and salt rejection. Neutrons, on the other hand, penetrate the whole membrane, thus characterizing pores and the fibers of the nonwoven fabric of radii between Å and several µm as well as identifying the polymers of the entire membrane. 

However, the analysis of asymmetric TFC–RO/NF membranes with SANS is complicated and work-intensive as the scattering is strong and locally not specified. These difficulties were largely resolved by corrections for multiple scattering and by performing contrast variation measurements, which allow for identification and a more detailed morphological characterization of the membrane polymers. Scattering from the PA skin layer of the membrane is almost non-detectable with SANS due to its small thickness in comparison with both supporting layers. Only at large scattering angles analogous to large **Q** the morphology of the PA layer might become visible when scattering from pores of several Å size are dominating. PALS supports the existence of such pores as well as a combined study of SANS and PALS on a standalone PA skin layer in Ref. [4].

An important motivation of our effort is to demonstrate the strength of a joint exploitation of SANS and PALS for revealing the bulk morphology of RO and NF membranes. This effort provides a basis of interpretation for our operando RO–SANS desalination experiments including membrane compaction, membrane scaling, and biofouling as well as concentration polarization in the presence of organic molecules in a simulated secondary effluent (SSE) solution [5,6,7] or aqueous silica solution. Some of the very first results of those operando RO–SANS experiments are reported in [8].

## 2. Materials and Methods

### 2.1. Thin Film Composite Membranes

Six commercial TFC membranes with different nonwoven support layers are listed in Table 1. The producer of the RO98 pHt membrane claims a sodium chloride rejection of ≥ 98% and a tolerance of high pH and temperature. The Dow Filmtec RO membranes SW30HR, BW30LE, and XLE are, respectively, optimized for high rejection seawater, low energy brackish water, and extra low energy brackish water RO desalination. These membranes are composed of, respectively, polyamide, polysulfone, and polyester of 0.2 µm, 40 μm, and 120 μm thickness. The producer recommends the NF270 membrane for removing organic carbon (TOC) and trihalomethanes (THM) from surface and ground water while maintaining high salt passage and partial hardness removal. The TM820 seawater desalination membrane consists of fully cross-linked aromatic polyamide composites.

The XLE membrane was grafted with methacrylic acid (MA) by redox-initiated graft polymerization in order to decrease biofouling propensity as previously described [9]. Grafting of RO membranes with MA is an example of surface modification for reducing fouling propensity. This strategy is based on surface hydrophilization of the membrane, thereby acquiring low adherence of organic substances and biological molecules, resulting in lower organic- and biofouling of polymeric membranes in water treatment processes [10,11]. The grafting of XLE membranes with MA was carried out by immersing the membranes in an aqueous MA solution followed by adding potassium metabisulfite (K_2_S_2_O_5_) and potassium persulfate (K_2_S_2_O_8_) [9]. The solution with the membrane was agitated gently on Unimax 1010 orbital shaker (Heidolph, Kelheim, Germany) for 20 min. The reaction was stopped by discarding the solution and washing the membrane rigorously with water.

The TM820 membrane was immersed for 1–5 days in a solution that simulated desalination of treated domestic wastewater at a stage of 80% recovery [5]. Then, the membranes were gently rinsed with 50 vol % ethanol/water, and dried.

### 2.2. Small-Angle Neutron Scattering (SANS) Instruments

The neutron experiments were performed at two SANS instruments (KWS 1 and KWS 3), both operating at the MLZ, Garching, Germany [12], covering a **Q** from 10^−4^ to 0.2 Å^−1^, thereby allowing the detection of pores of Å to µm in radius. Most experiments were performed at KWS3, the instrument for very small scattering angles (VSANS) and only part of the experiments was performed at the classical pin-hole instrument, KWS 1 (SANS), covering a range between 10^−3^ to 0.4 Å^−1^. The modulus of ***Q*** is determined according to Q=4π/λsin(δ/2) from the scattering angle (δ) and the neutron wavelength (λ). The scattering vector is proportional to the change of neutron momentum p=ℏQ (de Broglie relationship) and its modulus indicates the range where objects of 1/Q dimension mainly contribute to scattering. 

Experiments detecting neutrons scattered at very small angles (KWS 3) became possible by implementing a high-quality elliptical mirror focusing the neutrons onto the detector mirroring a few mm^2^ large area of the aperture at the entrance of the instrument with only negligible contributions from outside this area [13,14]. The detector was 9.50 m from the sample position and λ = 12.8 Å with a spread in wavelength of Δλ/λ = 20%. The experiments at KWS 1 were performed with λ = 7 Å (Δλ/λ = 10%) and a variable sample to detector distance between 20 to 1.5 m. Appendix A1, Appendix A2 and Appendix A3 summarize the necessary scattering laws for the analysis of the scattering patterns.

As already mentioned, the scattering pattern of neutrons comprises the morphological information of the entire membrane, which makes it challenging to distinguish between the contributions of the individual layers. This difficulty can be resolved when exposing the same membrane with a liquid contrast medium, whose solvent coherent scattering length density (ρ_S_) can easily be changed. A standard contrast medium is the mixture of H_2_O and D_2_O of varying composition, and lesser known examples are supercritical fluids (SCFs) such as CO_2_ and/or CD_4_ at variable pressure; we explain these in detail in Appendix A3. 

Table 2 presents parameters of the membrane polymers such as coherent scattering length density (ρ) and incoherent scattering cross-section (dΣ/dΩ_inc._) needed for analyzing the scattering data. As discussed in the theoretical section of Appendix A1 and Appendix A2 as well as in [15], the scattering length density (ρ) determines the strength of scattering. It is defined as the sum of the neutron coherent scattering length of the atoms (b_j_) forming the molecule, divided by the molecular volume (Ω_M_), that is $\rho =\sum_{j}b_{j}/\Omega_{M}$. The incoherent scattering cross-section (dΣ/dΩ_inc._) describes the inherently non-coherent part of the neutron scattering, is of equal strength in all directions and is considered as (uninteresting) background. All pore radii errors calculated from SANS data reflect only the statistical error of the fitting routine. 

### 2.3. Positron-Annihilation Lifetime Spectroscopy (PALS) 

Positron-annihilation lifetime spectroscopy (PALS) is a well-established technique for the analysis of the free volume in polymers (e.g., [19]). In polymers, the lifetime of ortho-Positronium (o-Ps), a bound state of positron and electron, is correlated with the pore size that o-Ps annihilate by interacting with surrounding electrons (pick-off process). The relation between the lifetime (τ) of o-Ps and the pore radius is described in the Tao–Eldrup model (see Appendix A4 and Equation (A11)). Within this model the pore is approximated by a sphere with an outer radius R_0_, and an inner radius R with a zone ΔR = R_0_ − R in which the electron density is not zero. 

A pulsed mono-energetic positron beam of variable energy allows a depth resolved determination of the pore size of amorphous matter. The positron implantation profile in the material characterized by the mean implantation depth can be chosen by varying the positron energy [20] of the pulsed low energy positron system (PLEPS) [21,22] operated in vacuum at the neutron-induced positron source at the MLZ (NEPOMUC) [23]. The mean implantation depth ($\bar{z}$) for a given positron energy (E) can be approximated by Equation (A12). 

Positron lifetime spectra containing more than 4 × 10^6^ counts were recorded as functions of implantation energy. The resolution function was obtained measuring a p-doped silicon carbide (p-SiC) reference sample with a well-known positron lifetime. This was followed by numerical deconvolution of the recorded spectra and fitted with a least-squares method based on the Levenberg–Marquardt algorithm. The fit residuals and the chi-square (χ²) value show reasonable results, if three lifetimes (τ_1_, τ_2_, τ_3_) were considered in the fit routine. The lifetimes τ_1_ and τ_2_, which are in the 100 ps range, are related to the direct positron annihilation and decay of para-Ps, whereas the longest component τ_3_ is associated with the above-mentioned pick-off lifetime of o-Ps in polymeric matter. Reasonable fits were only obtained for several spectra if considering an additional longer lifetime component with τ_4_ > τ_3_—see Appendix A4.

The statistical error bars for the lifetimes are small—typical in the order of 1–2% due to good counting statistics. Systematic errors are larger due to assumptions such as spherical shape of the pores or the uncertainty in the definition of pore radius seen by positrons. In consequence, we did not provide error bars for the absolute radii from PALS.

## 3. Results and Discussion 

**Figure 2:**
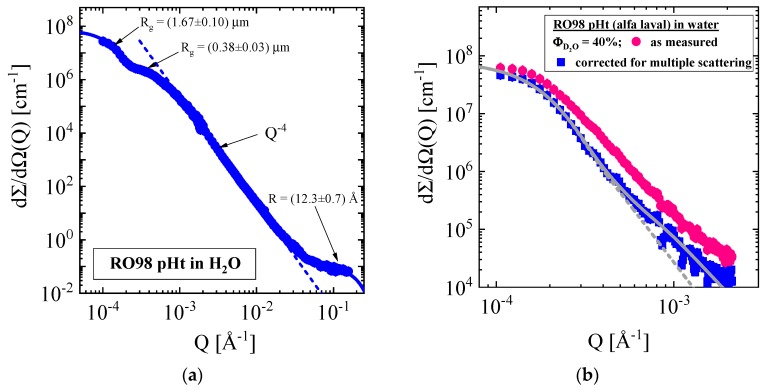
(**a**) Scattering pattern of the RO98 pHt membrane exposed to H_2_O after correction for multiple scattering. (**b**) Visualization of the effect of multiple scattering at small Q. Solid and dashed lines represent a fit of the scattering law (Equation (A4)). Contrast medium is water composed of 40 vol % D_2_O.

A first impression about the characteristics of scattering was gained for the Alfa Laval (RO98 pHt) membrane in Figure 2a. These data were measured at KWS3 and KWS1 together covering a Q interval from 10^−4^ to 0.2 Å^−1^ and are corrected for multiple scattering as outlined in Appendix A2. Similar scattering data over the same Q range were already presented for the XLE membrane in Figure 7 of a previous article [8]. The main differences between the Alfa Laval and Dow Filmtec membranes are, respectively, the overall thickness of 300 μm and 140 μm as well as the nonwoven fabric layers made from polypropylene and polyester fibers. Three groups of scattering entities of the order of 1.6 μm, 0.34 μm, and 12 Å for the radius of gyration (R_g_) become visible from the characteristic shape of the scattering pattern, which later will be attributed to the layers of the RO membrane. It should be kept in mind that scattering in the Q range below ~10^−2^ Å^−1^ is dominated by the two supporting layers. Scattering from the active skin layer could become visible beyond 5 × 10^−2^ Å^−1^ from scattering centers of about 12 Å radius, which was fitted with the form factor of spheres (Equation (A3)) in a Q range from 0.04 to 0.3 Å^−1^ considering the upturn (Q^−4^ Porod law) of scattering from the pores of the support layer.

The main focus of the present section is the identification and analysis of the two μm large scattering centers achieved from SANS contrast variation and from fitting the corresponding scattering laws. The subsequent section will discuss order of Å large pores observed with PALS mainly in the polyamide surface layer and with SANS at large Q.

Before getting to that point, we demonstrate the effect of multiple scattering in Figure 2b comparing the as measured with the on basis of the Equation (A7) corrected scattering pattern of the RO98 pHt membrane exposed to 40 vol % D_2_O aqueous mixture. Despite the membrane thickness of only 300 μm, multiple scattering becomes considerable because of the large scattering contrast of the medium with respect to the polypropylene fibers (Table 2). We corrected all SANS data measured at low Q for multiple scattering.

### 3.1. Identification of the Scattering Centers from SANS Contrast Variation

#### 3.1.1. RO98 pHt Membrane with Polypropylene Nonwoven Support

**Figure 3:**
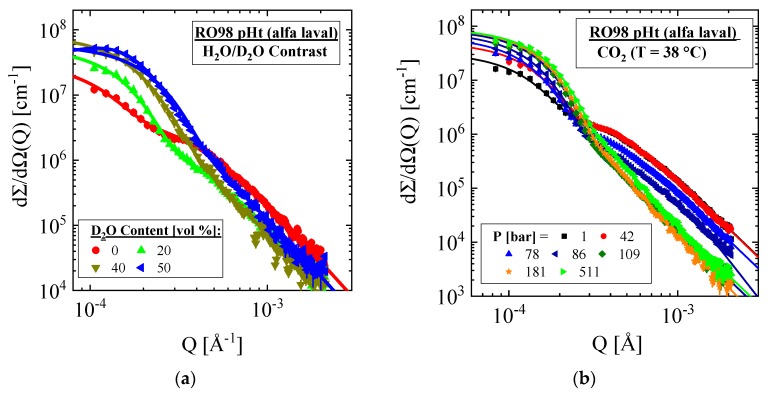
Scattering patterns of the RO98 pHt membrane at varying scattering contrast of the contrast medium (**a**) H_2_O/D_2_O and (**b**) SCF–CO_2_. Both contrast media show enhanced and reduced intensity with increasing D_2_O and CO_2_ concentration at small and large Q, respectively. The scattering curves were fitted with Equation (A4) shown as solid lines. All data are corrected for multiple scattering as outlined in Figure 2.

**Figure A1:**
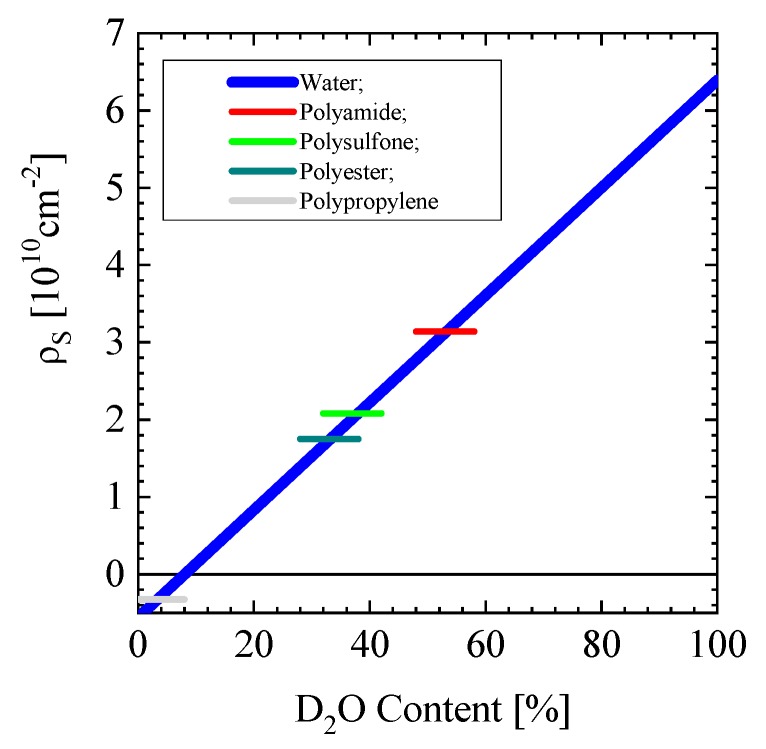
Coherent scattering length density of the H_2_O/D_2_O mixture according to ρ = () $-0.561+6.933{\times \Phi }_{D_{2}O}$ × 10^10^ cm^−2^ as function of D_2_O volume fraction. The coherent scattering length of the RO-membrane polymeric components are marked as short lines indicating the corresponding matching conditions.

**Figure A2:**
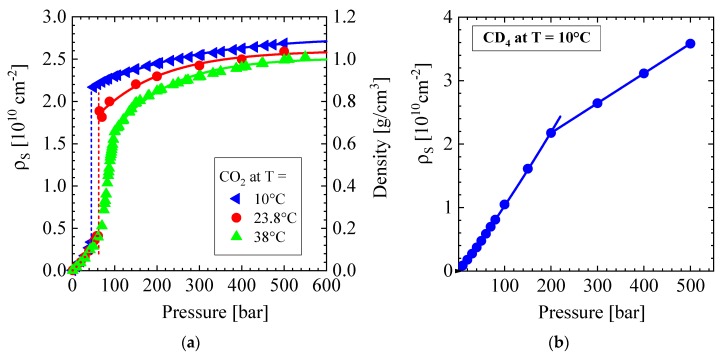
Scattering length density of CO_2_ (**a**) and CD_4_ (**b**) as calculated from scattering length and number density [15,24]. CO_2_ and CD_4_ become supercritical above their critical temperature and pressure at 31 °C/73.8 bar and −82.6 °C/45.9 bar, respectively.

Contrast variation experiments of the RO membranes were performed in the lower Q regime of the VSANS instrument showing enhanced scattering of µm large scattering centers. Scattering curves of the Alfa Laval membrane are depicted in Figure 3a,b for various scattering contrasts affected by the mixture of H_2_O/D_2_O and the SCF–CO_2_ at 38 °C. Figure A1 and Figure A2a show the corresponding scattering length densities (ρ_S_) of both media. Comparing the scattering data in Figure 3 allows us to observe the advantage of SCFs as contrast media where much finer tuning of ρ_S_ is achieved for the same membrane and only one membrane sample is required. On the other hand, when D_2_O–H_2_O mixtures are used as the contrast medium, an individual piece of membrane is required for each composition in order to make sure that the membrane is in equilibrium with the correct D_2_O–H_2_O composition. The main disadvantage of SCF is its smaller contrast range.

**Figure 4:**
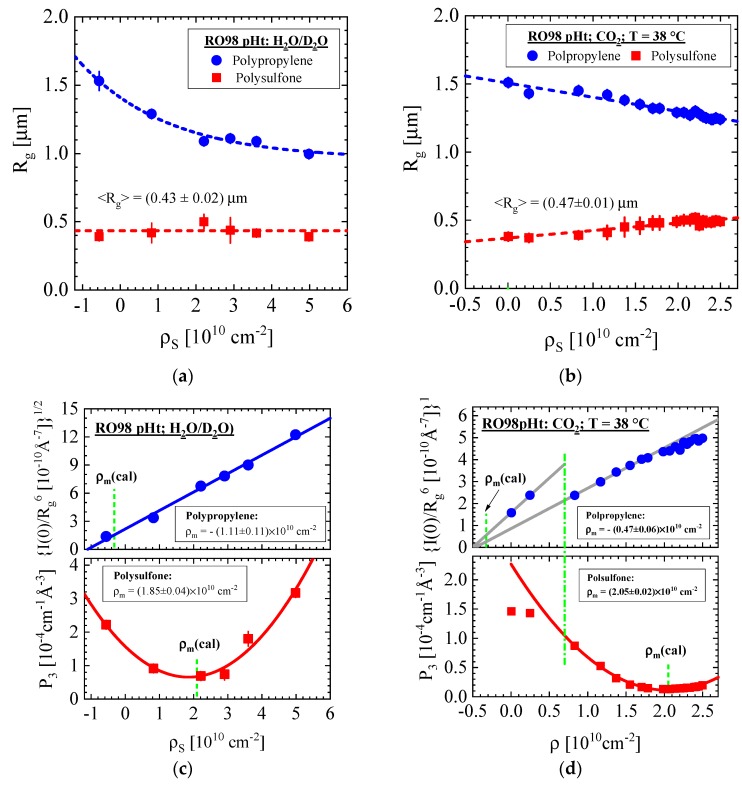
The small-angle neutron scattering (SANS) parameters of the RO98 pHt membrane are plotted versus ρ_S_ of the contrast media (**a**,**c**) H_2_O/D_2_O and (**b**,**d**) supercritical fluid (SCF)–CO_2_. The large and small pores are attributed to the polypropylene nonwoven fabric and to the polysulfone porous support layer, respectively. (**d**) The dashed–dotted line represents the gas/SCF phase boundary of CO_2_ at 38 °C. The symbol I(0) was chosen instead of dΣ/dΩ(0) because of space limitation.

The two scattering centers of larger and smaller size already observed in Figure 2a show an individual behavior, namely an increasing and declining intensity with increasing scattering contrast. Both scattering centers were fitted with Equation (A4) whose superposed scattering intensity, i.e., $d\Sigma /d\Omega \left(Q\right)=\sum_{i=1,2}{d\Sigma /d\Omega }_{i}\left(Q\right)$, is depicted as solid lines showing excellent agreement with the experimental data. The fit parameters of the two classes of scattering centers are plotted in Figure 4 versus ρ_S_ of the contrast medium and are compiled for all membranes in Tables 3 and 4. The size of the scattering centers, i.e., their R_g_, is depicted in Figure 4a,b versus ρ_S_ of the contrast media H_2_O/D_2_O and SCF–CO_2_. The parameters in Figure 4c,d are proportional to the scattered intensity at Q = 0 and thereby to the scattering contrast Δρ^2^ = (ρ_P,i_ − ρ_S_)^2^ of the membrane polymer (ρ_P,i_) and the contrast medium. The smallest intensity in case of ρ_m_ = ρ_S_ (see vertical line) refers to the type of polymer via comparison of ρ_m_ with the calculated ρ in Table 2. The polymers polysulfone and polypropylene are clearly identified as substances for the porous support and nonwoven fabric layers, respectively. The two parameters dΣ/dΩ_i=1_(Q = 0) and P_α__,i=2_ (α = 3) in Equation (A4) were chosen for identification in the large (i = 1) and small (i = 2) particles, respectively, because both parameters are minimally influenced by each other in their corresponding Q range. dΣ/dΩ_i=1_(Q = 0) and the amplitude P_α__,i=2_ (α = 3) dominate, respectively, at small and large Q by orders of magnitude.

An interesting observation of the nonwoven fabric layer is the continuous decline of R_g_ with enhanced ρ_S_, whereas R_g_ (pore size) of the polysulfone layer stays constant with H_2_O/D_2_O and shows only a slight enhancement of up to about 25% when exposed to SCF–CO_2_ at increasing pressures. The intensity parameter of the nonwoven material is plotted as $\sqrt{d\Sigma /d\Omega \left(0\right){/R}_{g}^{6}}$ versus ρ_S_ delivering a straight line, which becomes zero at negative ρ_m_. (upper part of Figure 4c,d). This combination of SANS parameters had to be chosen because of the large variation of R_g_ and its dependence of $d\Sigma /d\Omega \left(0\right)\propto N_{P}V_{P}^{2}\propto N_{P}R_{g}^{6}$ ($\Phi_{P}=N_{P}V_{P}$) (Equation (A2)). A constant pore number density (N_P_) was assumed. Matching at ρ_m_ occurs for the negative values of −1.11 and −0.47 in units of 10^10^ cm^−2^ in good agreement with the theoretical value for polypropylene (−0.325 × 10^10^ cm^−2^; Table 2). The amplitudes of P_3_ are 1.85 and 2.05×10^10^ cm^−2^ at ρ_m_ corresponding to the evaluated ρ = 2.08 × 10^10^ cm^−2^ of polysulfone in Table 2 (Lower part of Figure 4c,d). SANS contrast variation identified both polymers in compliance with the manufacturer’s information.

The “intensity” parameter of the fibers of the nonwoven fabric layer shows two distinctively different slopes below and beyond ρ_S_ = 0.72 × 10^10^ cm^−2^ (upper part of Figure 4d), which is identified as the CO_2_ gas/SCF phase boundary at 38 °C and 74 bar (Figure A2 and [24]). We ascribe this observation to the degree of wetting caused from capillary forces of the CO_2_ solvent and the fibers. The distinct slopes and amplitudes of the “intensity” parameter results from preferential wetting of the CO_2_ gas thereby reducing the scattering contrast (Δρ^2^). These considerations are consistent with the lower amplitude of P_3_ in the CO_2_ gas phase in comparison with the extrapolated P_3_ from the SCF phase. The finite scattering of the polysulfone layer at the matching condition is another interesting observation. We interpret this observation from pores and/or some other heterogeneity inside the fiber the contrast medium is not able to fill. We will discuss this issue later in context with the other membranes, which opens a relevant access to the internal morphology of membranes not being involved in the filtering process. 

#### 3.1.2. SW30HR Sea Water RO Membrane with Polyester Nonwoven Support Layer

**Figure 5:**
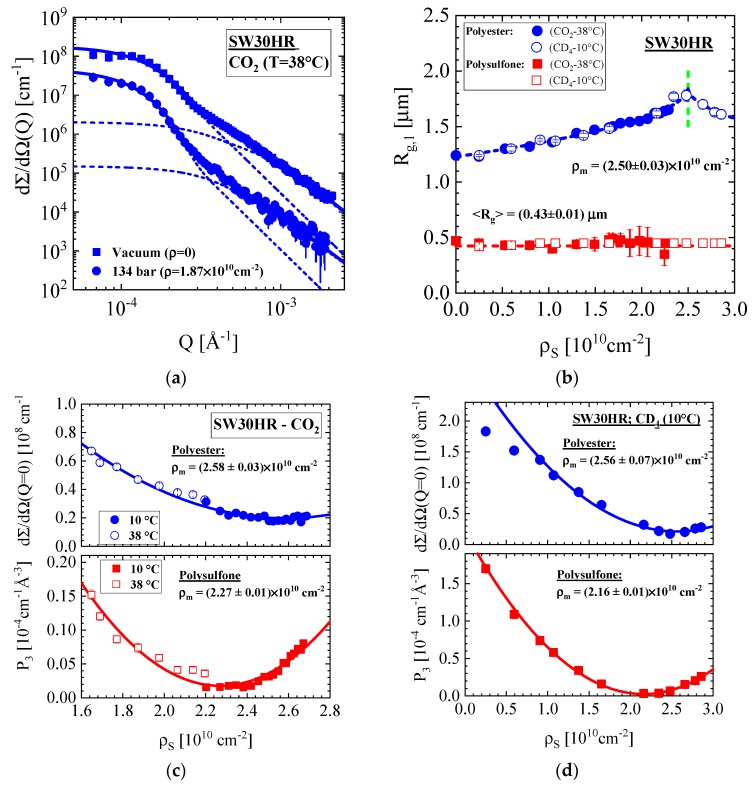
(**a**) Scattering from the SW30HR seawater RO membrane in vacuum and exposed to SCF–CO_2_ at 38 °C and 134 bar; (**b**) R_g_ of the polysulfone and polyester layer versus ρ_S_. (**c**,**d**) dΣ/dΩ(0) of the polysulfone and polyester layer versus ρ_S_ with the contrast media: (**c**) SCF–CO_2_ along the isothermal pathways at 10 and 38 °C and (**d**) CD_4_ at 10 °C.

Figure 5 shows contrast variation experiments with the contrast media SCF–CO_2_ at 10 and 38 °C and SCF–CD_4_ at 10 °C (see Figure A2b). Figure 5a shows two characteristic scattering patterns for the membrane in vacuum and exposed to SCF–CO_2_ at 38 °C and P = 134 bar corresponding to ρ_S_ = 0 and 1.87×10^10^ cm^−2^, respectively. The dashed curves represent fits of the individual scattering centers from both supporting layers. The larger R_g_ increases with ρ_S_ by about 25% whereas the smaller R_g_ stays constant (Figure 5b). The “intensity” parameters in Figure 5c,d deliver values of ρ_m_ in consistence with the polyester polyethylenterephthalat (PET) and polyethersulfone (PES) for the nonwoven and porous support layers, respectively. R_g_ of the nonwoven material in Figure 5b shows a small hump at ρ_S_ = (2.5 ± 0.03) × 10^10^ cm^−2^, which is in correspondence with the matching value ρ_m_ and is therefore identified as the radius of gyration (R_g,cl_) of “closed” heterogeneities not accessible for the contrast medium. We fitted the experimental R_g,1_ with the phenomenological equation $R_{g}\left(\rho_{S}\right)=A+B\times {\left(\rho_{m}-\rho_{S}\right)}^{\beta }$ with β = 0.5, A = (1.85 ± 0.02) μm, B = −(3.95 ± 0.11) × 10^−6^ μm × cm^2^^β^ shown as a dashed line. On basis of Equation (A8) we obtain R_g,op_ = (1.07 ± 0.03) μm and R_g,cl_ = (1.82 ± 0.04) μm for the nonwoven fabric morphology achievable and not achievable for the contrast medium, respectively. 

#### 3.1.3. BW30LE Low Energy Brackish Water RO Membrane

**Figure 6:**
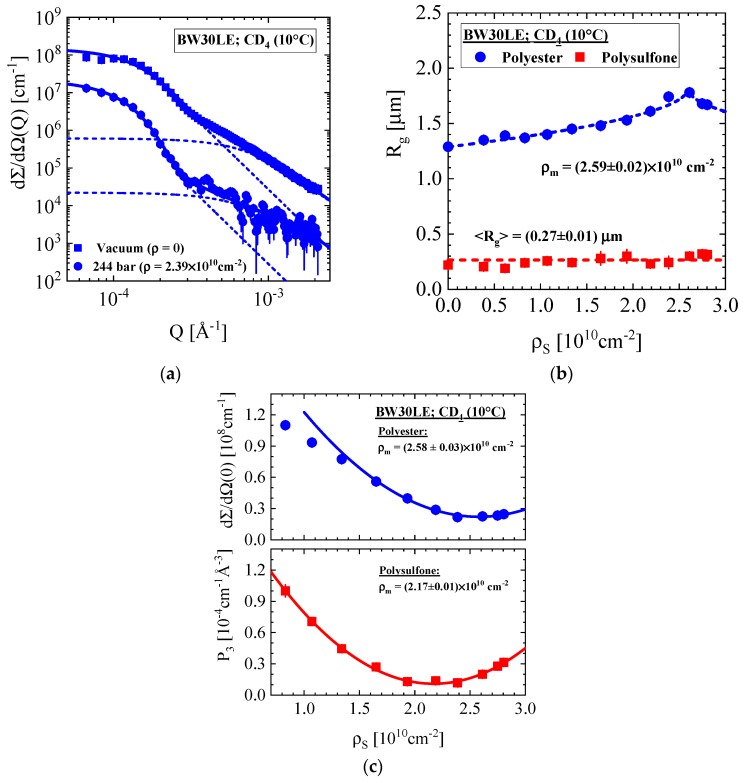
(**a**) Scattering pattern of the BW30LE RO membrane in vacuum and exposed to CD_4_ at 10 °C and P = 244 bar. (**b**) R_g_ of the nonwoven and porous layer versus ρ_S_ (**c**) The scattering intensity at Q = 0 of the polysulfone and polyester layer versus ρ_S_.

Figure 6 shows the scattering patterns of the BW30LE membrane. The two scattering patterns in Figure 6a represent the membrane in vacuum and exposed to SCF–CD_4_ at 10 °C and 244 bar, which causes a strong decline of scattering. The dashed curves represent the SW30HR membrane for the individual scattering centers of both layers. Figure 6b depicts the R_g_’s versus ρ_S_. The larger R_g_ shows a similar variation as the SW30HR membrane with a peak at ρ_m_ = (2.59 ± 0.02) × 10^10^ cm^−2^ but for the polysulfone layer a factor of about two smaller R_g_. The “intensity” parameters (Figure 6c) deliver ρ_m_ values consistent with ρ evaluated for the polyester PET and PES of the nonwoven and porous support layers, respectively. Again, R_g_ shows a weak hump at the matching condition of the nonwoven fabric layer. We achieve R_g,op_ = (1.17 ± 0.02) μm and R_g,cl_ = (1.82 ± 0.02) μm from a fit with β = 0.5 applying the same procedure as in the last section. It should be mentioned that the pore size of the polysulfone support layer is about 40% smaller (averaged value: R_g_ = (0.27 ± 0.01) μm) than that for the SW30HR membrane.

#### 3.1.4. Pristine and Grafted XLE BWRO Membrane 

**Figure 7:**
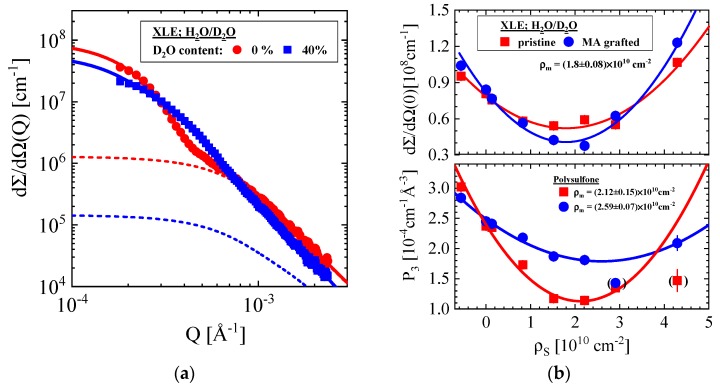
(**a**) SANS patterns of the pristine XLE membrane in H_2_O and in a mixture of H_2_O/D_2_O with 40 vol % D_2_O [8]. (**b**) Intensity parameters of pristine and grafted XLE versus ρ_S_. The upper and lower parts show the parameter of the nonwoven and polysulfone layer of the membrane, respectively.

Figure 7 shows the SANS results of a pristine and MA grafted XLE membrane. The contrast medium was H_2_O/D_2_O. Figure 7a shows two scattering patterns of the pristine membrane exposed to H_2_O and to an aqueous mixture of 40 vol % D_2_O showing the change of the larger and smaller scattering centers with the contrast medium. The dashed lines represent the fits of the smaller scattering centers. Figure 7b depicts the intensity dependent parameters plotted versus ρ_S_ similarly as for the other membranes. We find differences for the nonwoven material with respect to the other RO membranes: A constant (i.e., independent from ρ_S_) and smaller R_g_ = (0.91 ± 0.04) μm as well as a smaller ρ (ρ_m_ = (1.80 ± 0.08) × 10^10^ cm^−2^) were determined, suggesting a different fiber material. The pristine porous support layer shows a constant R_g_ of (0.23 ± 0.02) μm and a matching value of ρ_m_ consistent with polysulfone. The porous support layer is considerably influenced by MA grafting, showing a slightly larger R_g_ of (0.28 ± 0.01) μm, a 20% larger ρ_m_ and an appreciably larger intensity at ρ_m_. The graft polymerization of the RO membrane occurs on the active skin layer as well as inside the porous support. Hence, poly methacrylate partially fills the pores of the PES layer, thereby showing a smaller volume fraction of pores achievable for water. The larger R_g_ might result from the averaging process (z-average) of the pore size distribution, i.e., stronger weighting of the larger pores. MA grafting seemingly prefers filling the smaller pores in the bulk of the porous support layer. 

### 3.2. Discussion of the SANS Data from the Supporting Layers 

The relevant SANS parameters of the nonwoven fabric and porous support layers are compiled below in Tables 3 and 4. As already mentioned, scattering from the polyamide surface layer is too weak to be detected in the Q range of less than 10^−3^ Å^−1^ (Figure 3, Figure 4, Figure 5, Figure 6 and Figure 7 ) due to its thickness of less than one µm in comparison with the support layers. We, however, determined the morphology of 10 standalone polyamide layers of µm thickness with SANS and PALS as reported in [4]. 

#### 3.2.1. Nonwoven Fabric Support

The SANS parameters of the nonwoven fabric layer are compiled in Table 3. The scattering length density at matching condition (ρ_m_ in the fourth column) gives information about the polymer of the fibers, i.e., it identifies the polymer by comparing it with the evaluated ρ in Table 2. According to the producer’s information, the nonwoven fabric of the RO98 pHt membrane is made from polypropylene, showing a theoretical value of −0.325 (all values in units of 10^10^ cm^−2^, Table 2). The experimental ρ_m_ determined from SCF–CO_2_ contrast variation is −0.47, in good agreement with the calculated one, whereas the contrast medium water is −1.11, a worse result. A linear fit of the “water” data in the upper Figure 4c excluding the smallest parameter measured in H_2_O (smallest ρ_S_) displays a ρ_m_ of (0.88 ± 0.15), which is still too large. We speculate that SCF–CO_2_ has better wetting condition as water. The fibers of the nonwoven fabric layers of the SW30HR and BW30LE membranes were identified as polyethylenterephthalat (PET) in excellent agreement with its theoretical ρ = 2.58 × 10^10^ cm^−2^. On the other hand, the corresponding ρ_m_ of the XLE nonwoven fabric delivers a value of 1.80 × 10^10^ cm^−2^, which is too small to be identified as pure PET. Bi-component fibers composed, e.g., of 27 vol % low-density polyethylene (PE) and 73 vol % PET (see Table 3), would fit the SANS data. The remarkably improved tensile strength of such bi-component fibers is sometimes the preferred material for nonwoven fabric [25]. 

**Figure 8:**
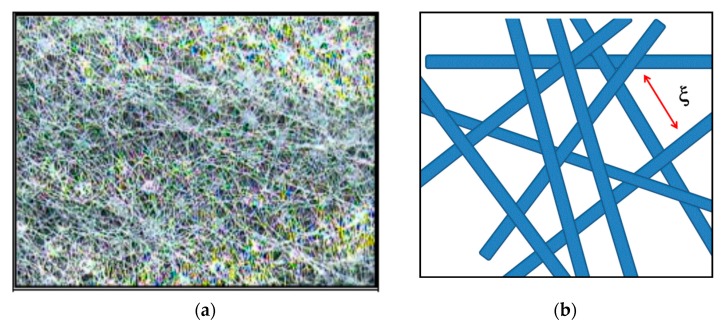
(**a**) Microscope view (40×) of nonwoven fabric of 12 g/m^2^ polyester (taken from p. 22 of ref. [25]) and (**b**) sketch of a fiber network with the average length ξ of the segment between two nodes of the network.

The scattering from the nonwoven fabric layers originates from centers of the order of µm in radius, i.e., R_g_ and volume fraction of ~2% (column 9 and 10). Polymer fibers are the minor phase of the nonwoven fabric material loosely packed with a large free space of ~98% in accordance with the Babinet principle [26] (p. 32). Figure 8a depicts a typical conformation and interplay of fibers of a polyester nonwoven fabric, schematically amplified in Figure 8b. The coherent scattering characterizes rods of length ξ and cross-section R, whose R_g_ is expressed as representing a solid rod of length ξ and cross-section radius R [26] (p. 159). The length ξ represents the average distance of the fibers between two nodes an interpretation, which has much in common with the blob model for semi-dilute polymer solutions [27] (Chapter III).

We observe two types of scattering centers, one affected by the contrast medium the other one not affected by the contrast medium. The first one represents the global fiber, whereas the second one internal heterogeneities of the fiber such as closed pores. Internal heterogeneities of fibers could be of different origin such as phases of crystalline and amorphous regions, closed pores, or binary phases as expected of bi-component fibers proposed for the XLE membranes. The ratio of scattering intensity from internal heterogeneities with respect to the total fiber is expressed as γ in the 7th column of Table 3 as evaluated from dΣ/dΩ(0) and P_3_ in Figure 4, Figure 5, Figure 6, Figure 7 for the nonwoven fabric and porous support layers, respectively at ρ = ρ_m_ and ρ = 0.

The volume fraction of the fibers and internal structure listed in the 9th and 10th column of Table 3 were derived from the second moment (Q2) of the intensity (Equation (A6)), the scattering contrast at ρ_m_, and multiplied with (1−γ) and γ (for definition of γ, see Equation (A10)), respectively. The integrals of Q2 were determined from the fitted scattering pattern of the polysulfone and nonwoven support layer as, for instance, shown as a dashed line in Figure 5a and Figure 6a. The choice of the fitted curve allows integration over larger Q. These parameters, however, represent values averaged over the total volume of the membrane due to absolute calibration of the scattered intensity. For the Dow Filmtec membranes, the volume fraction of the fibers Φ_fil_ has to be multiplied with 140/100 assuming a porous support layer of 40 μm thickness. Considering this correction, we find a fiber volume fraction between 2% and 4% which corresponds to 0.028–0.056 g/cm^3^ mass density of PET nonwoven fabric in fair agreement with values from literature of 0.019–0.035 g/cm^3^ for nonwoven fabric of polyester fibers in [28] (Table 2). In addition, the volume fraction Φ_int_ has to be divided by Φ_fil_ delivering for the SW and BW membranes a volume fraction between 17% and 21% if representing closed pores (Φ_cl_). 

Internal fiber heterogeneities could be of different origin. Crystalline and amorphous regions are always present in fibers due to the production process for improved mechanical strength as extensively studied with SANS [29,30,31]. The scattering contrast of crystalline/amorphous phases of PET fibers is with Δρ = 0.13 × 10^10^ cm^−2^ negligibly weak; a 50% volume fraction of crystalline–amorphous morphology would deliver a Q2 = 8.3 × 10^−6^ cm^−1^Å^−3^ which accounts for about 1% of the measured one. Therefore, we interpret the internal scattering of the fibers of the SW and BW membranes as from internal pores. In contrast, the determined large γ of the XLE membrane, i.e., their strong internal scattering, is consistent with the proposed bi-component composition of the fibers with the PET and PE polymers. The scattering contrast of PET and PE has been evaluated from the corresponding ρ in Table 2 as Δρ = 2.89 × 10^10^ cm^−2^ delivering a Q2 = 4.12 × 10^−3^ cm^−1^Å^−3^ for a 50 vol % PET and PE melt (Equation (A6)). After multiplication, this Q2 with the averaged fiber volume fraction of 1.7% gains 7.0 × 10^−5^ cm^−1^Å^−3^, in consistence with the experimental example of Q2 = 7.3 × 10^−5^ cm^−1^ Å^−3^, which was obtained after multiplication Q2 = 2.89 × 10^−4^ cm^−1^ Å^−3^ with γ = 0.65 (Table 3) and normalization to the correct scattering contrast (1.80 × 10^10^/2.89 × 10^10^)^2^. R_g_ of the XLE membrane is independent from ρ_S_ in contrast to the SW30HR and BW30LE membranes discussed in context in Figure 5b and Figure 6b. 

#### 3.2.2. Porous Support Layer

The SANS parameters of the porous support layer are compiled in Table 4. Except for the MA grafted XLE membrane, the scattering length density of the porous support layer of all membranes deliver an average value of ρ_m_ of (2.13 ± 0.05) × 10^10^ cm^−2^. This value better corresponds to the PSU than PES polymer according to the listed theoretical ρ in Table 2, confirming the producer’s information.

The listed intensity ratio γ is 44% larger for the RO98 pHt membrane when exposed to water at ambient pressure, whereas SCF–CO_2_ with γ = 5% fills the pores much better (95%), to the same degree as the SW30HR and BW30LE membranes. On the other hand, both XLE membranes show a larger degree of internal structure, i.e., pores not achievable for the contrast media. The MA grafting causes a ~20% enhanced ρ_m_ of 2.6×10^10^ cm^−2^, whereas the pore radii (R_g_) are constant within the given measurement error. The Dow Filmtec membranes show open pores with an average volume fraction of 4.2% when normalized to the total volume of the membranes. As these pores are part of the polysulfone support layer, the pore volume fraction has to be normalized to the thickness of the support layer (40 μm), i.e., has to be multiplied by the ratio of membrane thickness (140 μm) and support layer thickness (40 μm), i.e., 140/40 = 3.5, which delivers a pore volume fraction of nearly 15%, consistent with results of ref. [32] (Table 1), reporting a surface porosity (area coverage) between 5.9% and 14.5%. However, it should be stressed that SANS determines the volume fraction of pores in bulk. It should also be noted that the MA grafting is expected to partially fill the pores of the underlying support layer (see Figure 1, the PES side of the membrane) of the XLE membrane, since graft polymerization occurs both on the active skin layer and inside the porous support.

### 3.3. PALS Data

**Figure A3:**
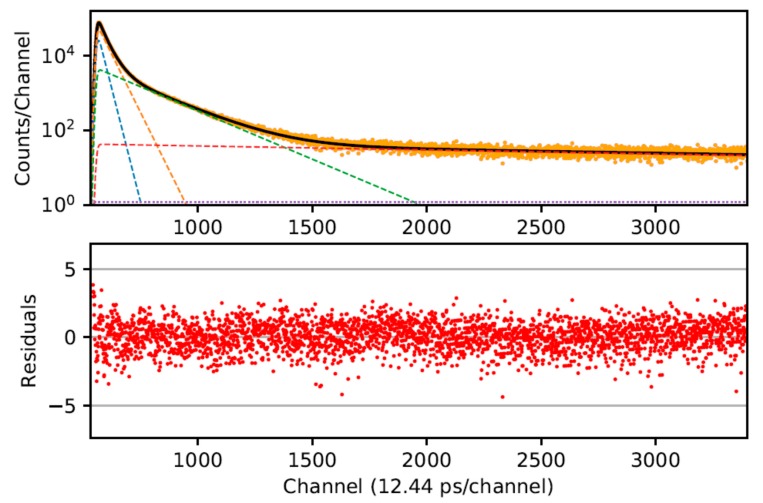
Typical PALS spectrum fitted with four lifetimes. The black line comprises four lifetime components (colored dashed lines) of a raw positron lifetime spectrum (dots) of the RO98pHt membrane recorded at a positron implantation energy of 8 keV.

The lifetime τ_3_ obtained by PALS estimates the mean size of pores on basis of the Tao–Eldrup model (Equation (A11)), which assumes o-Ps annihilation in spherically shaped pores of radius R [33]. The mean positron implantation depth is calculated according to Equation (A12) from the positron energy, assuming a mass density of 1.3 g/cm^3^ for the samples (Table 2). PALS determines pores of nanometer size at the position of the implantation depth, at distances up to about 4 μm from the membrane surface. This includes the polyamide skin layer as well as the adjacent part of the polysulfone layer. The intensity I_3_ is related to the total free volume in the polymer and allows observing changes of the free volume. The positron lifetime spectra were analyzed by minimizing the residuum between a fit comprising three to four lifetime components and the experimental data using the standard software PALSfit [34]. Figure A3 in the Appendix A shows a typical fit of a positron lifetime spectrum for the RO98 pHt membrane.

**Figure 9:**
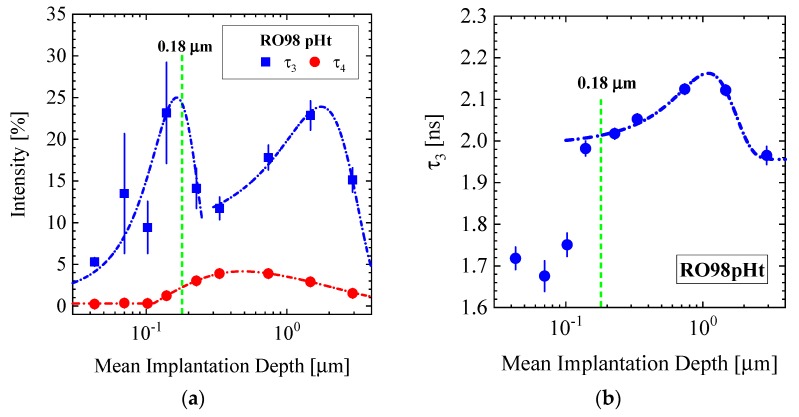
(**a**) Intensity of positron lifetimes τ_3_ and τ_4_ as well as (**b**) the lifetime τ_3_ of the RO98 pHt membrane versus mean implantation depth. The increases of intensity and lifetime itself are related to larger pore volume fraction and larger volume of pores, respectively. The appearance of the long lifetime τ_4_ (solid spheres in (**a**)) indicates a larger mean pore radius in the range of 40–90 Å. The green dashed line marks the interface between PA and support layer.

**Figure 10:**
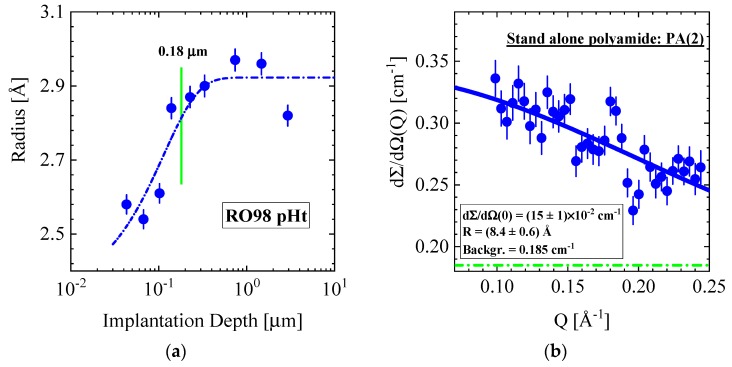
(**a**) Radius of pores in the RO98 pHt alfa laval membrane versus implantation depth as determined from τ_3_ in Figure 9b. Figure 2a shows the corresponding SANS data at large Q delivering a radius of (12.4 ± 0.7) Å. (**b**) For comparison, large Q data from a standalone polyamide layer are depicted delivering a radius of 8.4 ± 0.6 Å from fitting the form factor of spheres (these data are published in ref [4]). The blue line represents a fit of the data with Equation (A3) and the dashed–dotted line represents the background scattering.

Figure 9a,b shows the measured intensity and lifetimes in the cleaned RO98 pHt membrane. The cleaning procedure of the RO98 pHt membrane was standard: First, exposure of the membrane to 50% water (DI) and isopropanol solution, then to pure water (DI) both for 24 hours before drying took place for five days in fresh air. An additional longer lifetime τ_4_ appears in the lifetime spectra with values between 60 and 90 ns. These large lifetimes are beyond the Tao–Eldrup model. However, a mean pore radius in the order of 40–90 Å can be estimated considering the o-Ps mean free path and measurements at room temperature [33,35], which is consistent with the observation of individual voids of about 75 Å in the PA surface layer [36]. The near surface region (<600 Å) of the sample exhibits small free volume, which is indicated by the low intensity of both τ_3_, and τ_4_ (Figure 9a) and the decline of τ_3._ This is according to a smaller mean pore volume of around 60 Å^3^ (R = 2.5 Å) (Figure 10a) as will be discussed in Section 4. 

### 3.4. Pores of Nanoscale Dimension Determined from PALS and SANS 

#### 3.4.1. RO98 pHt Membrane 

We compare the results from PALS with the SANS data measured in a Q range between 0.1 and 0.4 Å^−1^. In this range, SANS becomes sensitive to pores of the order R = 10 Å. However, interpretation becomes difficult, as the localization of the scattering centers in the membrane is not possible and incoherent scattering becomes relevant, i.e., dΣ/dΩ_inc_ in Table 2.

Figure 10a shows the pore radius as a function of implantation depth of the cleaned RO98 pHt membrane. The pore radius increases continuously from 2.5 to 2.9 Å in the surface layer depth from 0.02 to 0.18 μm, before it becomes relatively constant in the measured membrane depth of 2.9 μm, thereby covering the active polyamide and part of the porous polysulfone support layer. 

The corresponding SANS data of the same membrane are shown in Figure 2a, whose large Q data are fitted with the form factor for spheres in Equation (A3) delivering dΣ/dΩ(0) = (0.12 ± 0.01) cm^−1^ and a R = (12.3 ± 0.7) Å, from which we evaluate Φ × Δρ^2^ = (0.15 ± 0.03) × 10^20^ cm^−4^ (Equation (A2)). For comparison, we depict in Figure 10b the large Q data from a standalone polyamide layer published in ref. [4]. The fit using the form factor for spheres delivered dΣ/dΩ(0) = (0.15 ± 0.01) cm^−1^ and a pore radius of R = (8.4 ± 0.6) Å yield a four times larger Φ × Δρ^2^ = 0.60 × 10^20^ cm^−4^ and a pore volume fraction (Φ) of 6.2%. In ref [4], we attributed the larger pore size of SANS to the strong correlation of the pores forming a fractal network structure. The scattering data of the RO98 pHt membrane and the standalone polyamide layer are in similar range even though the membrane data represent the entire membrane thickness.

#### 3.4.2. SW30HR, BW30LE, and NF270 Membranes

**Figure 11:**
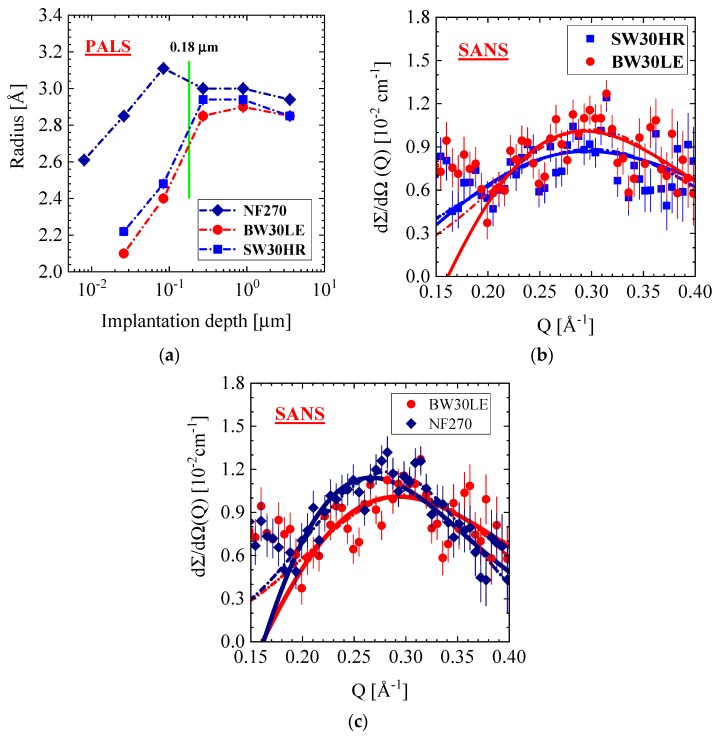
(**a**) Radius of the pores versus implantation depths for different membranes as determined with positron-annihilation lifetime spectroscopy (PALS). The symbols cover the error bars. (**b**,**c**) Large Q SANS data in vacuum from the same membranes showing an interference peak at Q_m_ = 0.30 (0.28) Å^−1^ from densely packed pores. The solid and dashed–dotted curves correspond to fits of the hard-sphere structure factor and Gaussian distribution, respectively.

In this section, we present PALS and the corresponding SANS data of the SW30HR, BW30LE, and NF270 membranes. Figure 11a shows the PALS data versus implantation depth. The BW membrane shows slightly smaller pores than the SW membrane but otherwise the same shape of increasing radii, which become constant (about 2.9 Å) beyond the implantation depth of 0.18 μm in consistence with the data of the RO98 pHt membrane. 

In this range of implantation, the NF270 membrane also shows pores of increasing, but overall ~30% larger size, which seems reasonable for a thinner active polyamide layer. Figure 11b,c display in the Q range from 0.15 to 0.40 Å^−1^ the corresponding SANS pattern of the SW30HR, BW30LE and NF270 membranes showing similar sensitivity to micro pores as PALS. For all three membranes, we observe an interference peak at around 0.29 Å^−1^, which corresponds to a periodicity length of the pores of about 25 Å (i.e., Λ_C_ = 1.23 × (2π/Q_C_); (see ref. [37], p. 73). Such an interference peak is not observed for the RO98 pHt membrane (Figure 2a). The scattering patterns in Figure 11 were fitted with two functions. The Gaussian distribution (dashed–dotted lines) according to $d\Sigma /d\Omega (Q)={d\Sigma /d\Omega (Q}_{C})\times \exp(-0.5\times {{((Q-Q}_{c})/\Delta Q)}^{2})$ was used for the determination of the peak position and width ΔQ = (Q_A_ − Q_C_) ($d\Sigma /d\Omega \left(Q_{A}\right)$ = exp (−0.5) = 0.607) and the structure factor was used for concentrated spheres (Equation (A5); solid lines) to determine size (R) and volume fraction (Φ) of the pores. The correlation length (η) of the ordering of pores can be derived from the half-width ΔQ of the Gaussian function according to $\eta =\sqrt{1.5}/\Delta Q$.

The PALS and SANS parameters are compiled in Table 5. At a membrane depth larger than 0.18 μm, PALS delivers an average pore radius of R = 2.9 Å for the RO and pores only a few percentages larger for the NF membranes in the PA and part of the polysulfone support layers. The fit of the SANS data with the Gaussian function delivers an average distance of the pores of Λ = (26 ± 1) Å, whereas Q2 (Equation (A6)) gives a pore volume fraction between 1.5% and 1.7% with respect to the total membrane volume. The fits of the same SANS data with the structure factor of concentrated spheres (Equation (A5)) account for pore radii, which are two to three times larger than the PALS values and show a volume fraction between 14% and 26%, the larger one for the NF membrane. The latter values of volume fraction were determined from the shape of the structure factor (Equation (A5)) and are thereby independent from absolute calibration of the scattered intensity. A comparison of these values with those from Q2 analysis allows an estimation of the effective thickness (d_eff_) of that part of the membrane containing the micro pores. Values of d_eff_ = 19 and 12 μm were estimated for the SW and BW membranes, whereas a smaller d_eff_ of 9 μm for the NF membrane.

Several PALS studies show pores of similar radii in the PA layer. For instance, ref. [38] states that "commercially available RO membranes have a mean free-volume hole-radius of 2.0–2.9 Å in the active skin layer with the thickness of approximately 1000 Å". In another study [39], it is determined by PALS that" the thin films of cross-linked aromatic polyamide RO membranes are composed of two types of pores having radii of about 2.1–2.4 Å from τ_3_ and 3.5–4.5 Å from τ_4_". Fujioka et al. [40] found that ESPA2 and ESPAB membranes have the same mean free-volume hole-radii of 2.89 Å, while that for SWC5 membrane was determined to be 2.59 Å. It was found that these numbers correlate with the rejection properties of the corresponding membranes.

Our PALS data show a gradual increase in pore size from about 2.1 to 2.8 and from 2.6 to 3.1 Å in a thin surface layer of 0.18 μm for the RO and NF membranes, respectively (Figure 10a and Figure 11a). To our knowledge, such a behavior of pore size is visualized here the first time for a wider set of commercial TFC membranes and seems to display a general property of TFC membranes. We have no adequate explanation. Nevertheless, the following discussion might shed some light on this phenomenon. As the PA skin and the PSU (PES) porous support layers are in glass-like states (see T_G_ in Table 2), we may consider the surface effects of polymer glasses. Physical aging, i.e., β relaxation was studied in poly(methyl methacrylate) (PMMA) glass in bulk and at the surface in ref. [41]. Physical aging drives glasses in the direction of larger mass density in accordance with the equilibrium state of the corresponding melt. The authors observed a reduced relaxation rate within a range of ~0.25 μm from the surface inward, becoming about two times smaller than the bulk relaxation rate. The reason for this behavior is still unclear according to the authors. However, there might be some correlation of continuously declining pore size towards the surface, accompanied with larger polymer mass density and the decline of the physical relaxation rate. 

Our last comment refers to the size and volume fraction of Å large pores. Whereas PALS shows almost no variation of pore size for the membranes in Table 5, SANS shows a larger variation. Figure 11b,c shows for the BW30LE membrane, respectively, a ~10% and ~50% larger pore radius and volume fraction, whereas for the NF270 membrane, the same pore radius but a ~86% larger volume fraction in comparison with the SW30HR membrane (Table 5).

#### 3.4.3. TM820 Seawater RO Membrane, Pristine and Scaled

**Figure 12:**
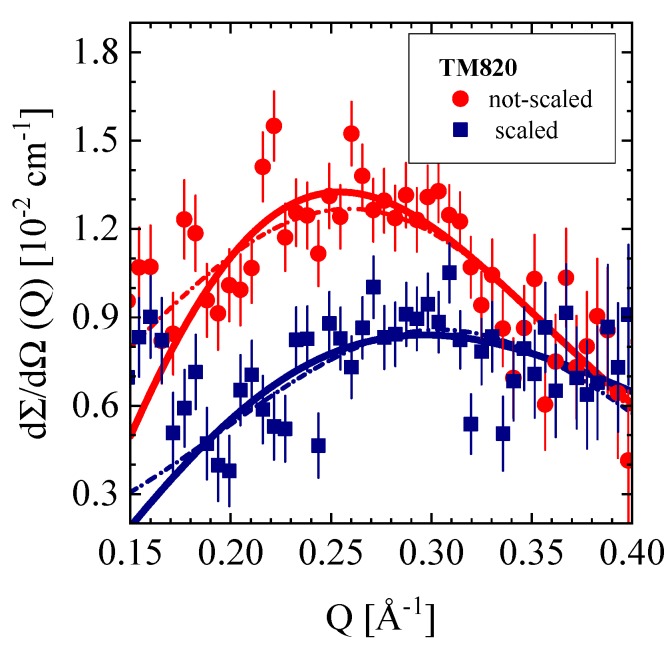
The hard-sphere model (solid lines) as well as the Gaussian distribution (dashed–dotted lines) describe the scattering from small correlated units, probably pores (Toray membrane company). Background scattering of 0.1 cm^−1^ was subtracted.

Finally, we present the large Q SANS data from the TM820 membrane in non-scaled and scaled conditions (see Table 1, Section 2.1 as well as Figure 12 and Table 6). A comparison of these data seems interesting as scaling shows a strong effect on micro pores, but no effect on the µm large pores, as the low Q data (not shown) do not show any visible effect from scaling. Pores of the radius of 1.8 Å and 0.9 Å were determined from PALS as compiled in Table 6, whereas SANS delivers densely packed pores of (7.6 ± 0.8) Å and (6.8 ± 2.6) Å radius distributed at the average distances of Λ = (30 ± 1.2) Å and (25.8 ± 0.9) Å in the unscaled and scaled membranes, respectively. The volume fraction of the pores in both membranes is about 18% and distributed in a ~16 μm thick layer of the membrane. It seems remarkable that scaling reduces the pore radii by a factor of two (PALS), whereas the SANS pore radius of the scaled membrane also seemed declined, however, this could only be determined with large error, whereas the volume fraction of pores declined between 10% and 20% as evaluated from Q2. 

## 4. Conclusions

In this manuscript, we reported about the morphology of several commercial RO membranes and a NF membrane from the perspective of SANS and PALS. Both techniques are complementary: PALS determines the depth profile of pores of radii of several Å, which are distributed over a distance of about 3 μm from the membrane surface. This includes the PA selective layer and the underneath supporting layer. SANS, on the other hand, allows identification of the membrane polymers and determination of pores of sizes from a few Å to μm dimension distributed over the entire membrane thickness. Identification of the membrane polymers and thereby porous support and nonwoven fabric layers of thin film composite (TFC), RO, and NF membranes needs the application of contrast variation experiments, in which the minima of the scattering length density (∝ ρ_S_^2^) identifies the polymer species via comparing with ρ of the polymers in Table 2. In this case, the scattered intensity should be zero, as observed for the polysulfone but not for the polyester layer, because of its internal heterogeneities not accessible to the contrast media. 

The polyamide skin layer of RO membranes is too thin to be detected at small Q by SANS. An independent SANS and PALS exploration of a standalone PA layer in ref. [4] delivered a network structure of pores of ~8 Å radius. Pores of similar size (Table 5 and Table 6) were detected for all membranes at Q > 0.1 Å^−1^, which might be allocated to the skin as well as to the porous support layer. On the other hand, PALS was able to detect pores with a radius of nearly 3 Å over a distance of nearly 4 μm from the membrane surface, thereby covering the active polyamide skin layer and part of the polysulfone porous support layer. The size of the pores seems to be unaffected by the transition from the polyamide to the polysulfone layer. An interesting observation is the declining pore size, up to 40% in a ~0.18 μm thick surface layer. All RO membranes depicted in Figure 10a and Figure 11a show similar shrinking within ~0.2 μm the same radius for pores, whereas the NF270 membrane in Figure 11a shows a smaller decline of less than 20%. This observation seems to be correlated with the glass state of the polymers, showing a reduced structural relaxation due to larger mass density at the surface layer, which is of similar thickness as outlined in ref. [41]. This correlation might be a valuable hint for better understanding surface effects in polymer glasses.

Freger [42] discusses an inhomogeneous mass distribution across PA skin layers of the RO membrane. He argues that PA skin layers are composed of negatively fixed and positively charged layers with an intermediate layer of larger polymer density acting as the actual selective barrier. As shown in [42] (Figure 6), this selective barrier is covered by a charged layer of ~1 μm forming the outer interface of the polyamide layer. Apart from a thickness of the PA layer that is too large, these calculations do not correlate with our PALS observation in Figure 10 and Figure 11a.

Pore radii in the PA membrane layer and the adjacent support layer as determined by PALS and SANS seem to differ as much as a factor of 2–3. The presented SANS radii are an average over eventual asymmetric pore dimensions. Furthermore, neglecting magnetic scattering, neutron scattering “sees” the nuclei of the surrounding matter. On the contrary, in PALS, the o-Ps looks for the nearest electron and thereby is sensitive to the shortest distance in the pore. Furthermore, Equation (A11) contains two different radii, the shorter hard-core radius R, assuming zero electron density and a shell of thickness ΔR = R_0_ – R = 1.656 Å to account for the increasing electron density towards the surface of the pore. The shorter radius (R) has been noted throughout this paper. Taking R_o_ as the pore radius instead, an average PALS radius of R_PALS_ = 4.5 Å is yielded at penetration depths of > 1.8 μm. This R_PALS_ is about 70% of the value determined by SANS, i.e., R_SANS_ = 6.1 – 6.8 Å. Taking into account that PALS “sees” the shortest distance within a pore, and SANS averages over all distances in a pore, both methods give very similar pore radii. A further aspect to be considered is the network structure of R < 10 Å pores predicted in [36] and observed with SANS/PALS in [4], which appears relevant for the transport of water. 

The polymers of the porous support and the fibers of the nonwoven fabric layers could be identified via contrast variation measurements. In the case of the porous support layer, polysulfone (PSU) and polyethersulfone (PES) polymers were identified, as well as open pores between 0.3 and 0.6 μm radius of a volume fraction of Φ ≅ 4.5 vol % accessible for the contrast media. The volume fraction of closed pores, inaccessible for the contrast medium, is less than Φ = 6 vol % except for the XLE membrane, showing a much larger Φ ≅ 50 vol %. Graft polymerization by MA on the XLE membrane shows a decline of accessible pore volume fraction due to the MA polymerization inside the pores, which decreases the volume of pores accessible for liquids. 

Segments of the fibers of the nonwoven fabric layer determine scattering, because of loose packing to a network as visualized in Figure 8b. Radii of gyration between 1 and 1.3 μm of polypropylene (PP) and polyethylenterephthalat (PET) between 2 vol % and 4 vol % were identified. Smaller fibers were observed for the XLE membranes whose chemistry could be explained by a mixture of 73 vol % PET and 23 vol % of low density polyethylene (PE). The latter observation is consistent with its large scattering from the internal structure of the fiber prepared from a mixture of two polymers. The other two membranes (SW30HR and BW30LE) show nearly 20% contribution from the internal structure of the fibers, which we interpret as from pores. The glass state of PET fibers may have some influence on scattering due to the internal structure, whereas polypropylene was in a condition of melt (T_G_ = −10 °C), which, however, cannot be tested with SANS due to its negative scattering length density (Figure 4c,d). 

## Figures and Tables

**Table 1:** Explored RO– and NF–TFC membranes.

Producer	Membrane	Type	Composition	Membrane Thickness [μm]	Experimental Technique
Alfa Laval ^1^	RO98 pHt	RO membrane	polyamide polysulfone-polypropylene	300	SANS/PALS
Dow ^2^ Filmtec	SW30HR	seawater high rejection	polyamide-polysulfone-polyester	140	SANS/PALS
	BW30LE	brackish water low energy		150	SANS/PALS
	XLE [8]	low pressure RO membrane		140	SANS
	NF270	nanofiltration membrane (NF)			SANS/PALS
Toray ^3^ (Japan)	TM820	sea water RO membrane			SANS

^1^ Thanks to Stephan Wild (Coordinator Environment) and Dipl. W.- Ing. Micha Kruse (Sales Engineer Service) from Alfa Laval Mid Europe GmbH in DE-21509 Glinde (Germany) for providing the RO membrane RO98 pHt; ^2^ The RO membranes SW30HR, BW30LE, XLE, and NF270 were provided from DOW FILMTEC (Minneapolis, MN, USA); ^3^ Seawater RO membranes TM820 were supplied from Toray Industries in Tokio (Japan).

**Table 2:** Relevant parameters of membrane material such as glass transition temperature (T_G_), incoherent scattering cross-section (dΣ/dΩ_inc_), coherent scattering length density (ρ), D_2_O volume fraction (Φ_match_) of water gaining the same ρ of the membrane polymer, i.e., to make it invisible for neutrons.

Molecule	Chemical Formula	Mass Density [g/cm^3^]	Molar Weight [g/mol]	Glass Transition Temp. T_G_ [°C]	Scattering Cross-Section dΣ/dΩ_inc._ [cm^−1^]	Scattering Length Density ρ [1010 cm^−2^]	Φ_match_ (D_2_O)
Aromatic Polyamide (PA) (Kevlar)	N_2_C_14_H_10_O_2_	1.44	238	60–75	0.233	3.10 ± 0.07	0.53
Polysulfone (PSU)	C_27_H_22_O_4_S	1.24	442.54	~220	0.237	2.08	0.38
Polyethersulfone (PES)	C_12_H_10_O_4_S	1.3–1.4	250.27		0.225	2.22	0.40
Polypropylene	C_3_H_6_	0.91	42.08	−10	0.50	−0.325	0.034
Polyethylenterephthalate (PET¸ Polyester)	C_10_H_8_O_4_	1.38 [16,17]	192.17	~79	0.37	2.58	0.45
PET (amorphous)		1.33 [16]			0.36	2.49	0.44
PET (crystalline)		1.52 [17]			0.41	2.84	0.49
Low density Polyethylene (PE)	C_2_H_4_	0.88	28.05	−125	0.48	−0.31	0.036
Methacrylic acid (MA) [18]	C_4_H_6_O_2_	1.015	84.83; (R = 3.23 Å)	-	0.27	1.12	0.24

**Table 3:** SANS parameters of the nonwoven fabric normalized to the thickness of the membrane. The parameter Φ_int_ represents the volume fraction of heterogeneities of the fibers as determined from the ratio of scattering at ρ = 0 and ρ_m_. (see Figure 5c,d, Figure 6c, or Figure 7b). The parameters of the RO98 pHt membrane were determined at sufficiently strong scattering contrast realized for the contrast media of water of 40 vol % D_2_O and SC–CO_2_ at 181 bar.

Membrane	Polymer	Medium	ρm [1010 cm^−2^]	dΣ/dΩ(0) [107 cm^−1^]	Rg [μm]	Ratio γ (Equation (A8))	Q2 [10−4 cm^−1^Å^−3^]	Φfil [vol %]	Φint [vol %]
RO98 pHt	PP	40 vol % D2O (Δρ = 2.68 × 1010 cm^−2^)	−(1.11 ± 0.11)	8.27 ± 0.22	1.10 ± 0.01	-	2.35	1.66	-
	PP	CO2 38 °C, 181 bars (Δρ = 2.07 × 1010 cm^−2^)	−(0.47 ± 0.06)	9.14 ± 0.2	1.30 ± 0.01	-	1.09	1.29	-
SW30HR	PET	Vacuum	2.59 ± 0.03	18.1 ± 0.6	1.25 ± 0.01	0.12	3.2	2.9	0.4
BW30LE	PET		2.58 ± 0.03	14.6 ± 0.5	1.26 ± 0.02	0.15	2.64	1.7	0.3
XLE	PET0.73/PE0.27		1.80 ± 0.08	8.08 ± 0.05	0.91 ± 0.04	0.65	2.89	1.6	2.9
Grafted XLE			1.79 ± 0.08	8.40 ± 0.06	0.94 ± 0.01	0.48	2.18	1.8	1.6

**Table 4:** Parameters obtained from contrast variation of the porous support layer (*) measured in vacuum or H_2_O). Φ×140/40 = 3.5 or 300/40 = 7.5.

	Polymer	Medium	ρ_m_ [1010 cm^−2^]	dΣ/dΩ(0) [106 cm^−1^]	R_g_ * [μm]	γ	Q2 [10^−4^ cm^−1^Å^−3^]	Φ_pore_ [vol %]	Φ_int_ [vol %]
RO98 pHt	PSU	H_2_O (Δρ = 2.36×10^10^cm^−2^)	1.85 ± 0.04	3.89 ± 0.05	0.43 ± 0.02	0.44	4.32	2.2	1.7
		Vacuum	2.05 ± 0.02	2.27 ± 0.01	0.47 ± 0.01	0.05	4.01	4.6	0.2
SW30HR	PES		(2.16 to 2.27) ± 0.01	2.04 ± 0.27	0.43 ± 0.01	0.02	4.06	4.3	0.1
BW30LE	PES		2.17 ± 0.01	0.70 ± 0.03	0.22 ± 0.02	0.05	4.62	4.7	0.3
XLE	PSU		2.12 ± 0.15	1.08 ± 0.02	0.23 ± 0.02	0.48	6.25	3.6	3.4
MA grafted XLE			2.59 ± 0.07	1.77 ± 0.05	0.28 ± 0.01	0.73	6.50	1.3	3.6

**Table 5:** Parameters obtained from PALS and SANS at large Q. The RO98 pHt and Dow-Chem. membranes were measured in H_2_O and air, respectively. Error bars for R determined by PALS and SANS reflect only statistical errors from the fitting routines.

Parameter	RO98 pHt	SW30HR	BW30LE	NF270
**PALS**				
R [Å] (at penetration depth > 0.18 μm)	2.88 ± 0.02	2.91 ± 0.05	2.87 ± 0.03	2.98 ± 0.04
**SANS—Gaussian Distribution**				
Q_C_ [Å^−1^]	-	0.30 ± 0.01	0.30 ± 0.01	0.28 ± 0.002
Q2 [10^−4^ cm^−1^ Å^−3^]	-	2.37	2.28	2.02
Φ_Q2_ [vol %]	-	1.72	1.66	1.47
	**Model of Spheres**	**Model of Hard-Spheres (Equation (A5))**		
dΣ/dΩ(0) [10,[10^−2^ cm^−1^]	12 ± 1	2.08 ± 0.10	2.64 ± 0.15	3.1 ± 0.1
R [Å]	12.3 ± 0.7	6.1 ± 0.5	6.8 ± 0.4	6.1 ± 0.2
Φ_0_ [%]	-	14 ± 2	21 ± 4	26 ± 2
Q2 [10^−4^ cm^−1^ Å^−3^]	2.33	2.6	2.53	2.3
Φ_Q2_ [vol %]	0.9–1.1	1.9	1.84	1.66
d_eff_ [μm]	-	19	12	9

**Table 6:** Parameters of non-scaled and scaled TM820 membranes determined from PALS and SANS at large Q.

Parameter	Pristine Membrane	Scaled Membrane
**PALS**		
R [Å]	1.82 ± 0.41	0.93 ± 0.84
**SANS—Gaussian Distribution**		
Q_C_ [Å^−1^]	0.26 ± 0.01	0.30 ± 0.01
Q2 [10^−4^ cm^−1^ Å^−3^]	2.69	2.16
**SANS—Model of hard-spheres**		
dΣ/dΩ(0) [10,[10^−2^ cm^−1^]	3.3 ± 0.2	2.0 ± 0.6
R [Å]	7.6 ± 0.8	6.8 ± 2.6
Φ_0_ [vol %]	18 ± 4	17 ± 7
Q2 [10^−4^ cm^−1^ Å^−3^]	2.78	2.50
Φ_Q2_ [vol %]	2.02	1.82
d_eff_ [μm]	~16	~14

## Appendix A

In Appendix A1 and Appendix A2 we provide the scattering laws applied for the analysis of the SANS scattering data, as well as an explanation of SANS contrast variation together with the scattering length densities of the contrast media H_2_O/D_2_O, and the SCFs CO_2_, and CD_4_, in Appendix A3. Appendix A4 outlines PALS in polymers.

### Appendix A1. SANS Scattering Laws

Scattering techniques using neutrons and photons are well established tools both from theoretical and experimental aspects [26] (p. 188). Scattering originates from domains that differ from their surroundings in their chemical composition and/or mass density. In our case, pores, or even segments of polymer fibers represent those domains. SANS is a quantitative method, which determines structural parameters, which are averaged over macroscopic large volumes of the order of 0.1 cm^3^. In this respect SANS is complementary to TEM, which makes individual domains visible.

The SANS pattern of the full membrane represents a linear superposition of scattering from all three layers weighted with the ratio of thickness according to d_i_/d_tot_ with “i” and “tot” indicating the polyamide, polysulfone, or polyester/polypropylene layer and the total thickness of the membrane, respectively. The differences in chemical composition and/or mass density are contained in the scattering contrast, which for neutrons is determined by the difference of ρ from the domain (P) and its surrounding (S) squared, i.e., ${\Delta \rho }_{i}^{2}={\left(\rho_{P,i}-\rho_{S}\right)}^{2}$. The scattering length densities of the relevant polymers are compiled in Table 2. The scattered neutrons intensity of layer “i” is expressed in Equation (A1) as differential macroscopic cross-section (dΣ/dΩ_i_(Q)) determined

(A1) $${d\Sigma /d\Omega }_{i}\left(Q\right)={d\Sigma /d\Omega }_{i}\left(0\right)\times F_{i}\left(Q\right)\times S_{i}\left(Q\right)$$

in units of cm^−1^ (i.e., scattering per cm^3^ of sample volume) as product of the form factor F_i_(Q) (F_i_(0) = 1), structure factor S_i_(Q) (S_i_(Q→∞) = 1), and macroscopic cross-section at Q = 0, which according to Equation (A2), is determined by the domain volume (V_P_), domain volume fraction (Φ_P_) of

(A2) $${d\Sigma /d\Omega }_{i}\left(Q=0\right)=\Phi_{P,i}V_{P,i}{\left[\rho_{P,i}{-\rho }_{S}\right]}^{2}$$

domains such as pores or fibers in layer i, and the scattering contrast ${\left[\rho_{P,i}-\rho_{S}\right]}^{2}$ compiled for relevant polymers of TFC membranes in Table 2. In cases of µm large pores we apply the form factor of spherically shaped domains of radius R showing the well-known expression in Equation (A3) [26,37], whereas for the larger pores we apply Beaucage’s equation (Equation (A4)) for

(A3) $$F(Q)=\frac{9{\left(\sin\left({\mathrm{QR}}_{P}\right)-{\mathrm{QR}}_{P}\cos\left({\mathrm{QR}}_{P}\right)\right)}^{2}}{{\left({\mathrm{QR}}_{P}\right)}^{6}}$$

quantitative analyses of the scattering pattern describing the data at Q < 1/R_g_ by Guinier’s approximation (first term) and at Q > 1/R_g_ by a power law ($P_{P}Q^{-\alpha }$) with the exponent α_i_ and the

(A4) $${d\Sigma /d\Omega }_{i}\left(Q\right)={d\Sigma /d\Omega }_{i}(0)\exp(-u_{i}^{2}{/3)+P}_{P_{i}}{\left[{\left(\mathrm{erf}\left(u_{i}^{2}/\sqrt{6}\right)\right)}^{3}/Q\right]}^{\alpha_{i}}$$

Amplitude P_P_ (second term) [43]. The radius of gyration R_g_ is defined according to $R_{g}^{2}=\int r^{2}\rho (r)dr/\int \rho (r)dr$ as second moment of the scattering length density ρ(r) being a function of the local vector r. [26] (p 158).The parameter u_i_ expresses the product of Q and R_g,i_.

The scattering law in Equation (A4) is valid in the limit of low particle concentration when scattering is determined from individual domains S(Q) = 1, i.e., there is no correlation between the particles. Scattering of higher particle concentration shows correlations such as those observed for micro pores in the polyamide layer formed as fractal structure [4] or, as shown in Section 3.4, for TFC membranes. The analytical expression of S(Q) in Equation (A5) describes an ensemble of

(A5) $$S_{\mathrm{HS}}(Q)=1-8\Phi \frac{3\left(\sin2\mathrm{QR}-2\mathrm{QR}\cos2\mathrm{QR}\right)}{{\left(2\mathrm{QR}\right)}^{3}}$$

concentrated spheres interacting via their excluded volume [26] (p. 172). The parameters, Φ and R, are volume fraction and radius of the spheres, respectively.

An important parameter is the second moment, $Q2=\int Q^{2}d\Sigma /d\Omega (Q)\mathrm{dQ}$, sometimes called the “invariant” of scattering of isotropic scattering centers, which according to Equation (A6) is determined only from the product of particle volume fraction and scattering contrast of the

(A6) $${Q2}_{i}={2\pi }^{2}\Phi_{i}\left(1-\Phi_{i}\right){\left[\rho_{P,i}-\rho_{S}\right]}^{2}$$

corresponding layer but does not depends on domain size [26].

### Appendix A2. Correction for Multiple Scattering

Correction for multiple scattering becomes relevant in case of low transmission of T < 0.3 in the classical SANS Q-range (Q > 0.001Å^−1^) or T < 0.8 for structures of µm in size measured in the extended Q-range of smaller than Q ≈ 10^−4^ Å^−1^ as measured with VSANS. A theoretical summary of multiple scattering has been given in refs. [44], which is based on original considerations by Schelten et. al. [45] (see also [46]). The numerical program for the calculus is found in ref. [47]. The essential formula for considering multiple scattering effects, Equation (A7), contains sample thickness *d*, neutron wavelength λ, and the apparently measured and corrected

(A7) $${d\tilde{\Sigma }}_{\mathrm{corr}}/d\Omega \left(r\right)=2\pi /\left(d\times \lambda^{2}\right)\times \ln\left[d\times \lambda^{2}/\left(2\pi \right)\times {d\tilde{\Sigma }}_{\mathrm{app}}/d\Omega \left(r\right)+1\right]$$

macroscopic cross sections ${d\tilde{\Sigma }}_{\mathrm{app}}/d\Omega \left(r\right)$ and ${d\tilde{\Sigma }}_{\mathrm{corr}}/d\Omega \left(r\right)$, respectively. The latter two are given in ‘real’ space connected via a Hankel transformation, i.e., for an arbitrary function a(Q) and the

(A8) $$a˜(r)=\int_{0}^{\infty }a(Q)\cdot Q\cdot J_{0}\left(\mathrm{Qr}\right)\mathrm{dQ}$$

Bessel function of zero order J_0_, if the scattering is isotropic. In practical terms, two transformations are necessary, first towards real space, deconvolution according to Equation (A7) and one back in reciprocal space to display the corrected scattering cross-section. Other proposed calibrations involve the explicit knowledge of the macroscopic cross sections for the incoherent scattering and the absorption. Practically, these methods did not provide better or more reliable results. For the proposed correction method here (Equation (A7)), this means that the apparent cross-section needs to be reliably calibrated. The Taylor expansion of Equation (A7) for small apparent macroscopic cross sections (i.e., ln (1 + ε) = ε − ε^2^/2 + ε^3^/3 − …) leads to degree wise corrections of multiple scattering corrections (i.e., 1, 2, 3 times), but they are not considered any further here.

### Appendix A3. The Importance of the Contrast Media for SANS Evaluation of TFC Membranes

The use of different contrast media for SANS makes it possible to identify scattering from the supporting layers of TFC membranes. In practice, the membranes are exposed to a series of solvents, whose scattering contrast, ${\Delta \rho }^{2}={\left[\rho_{P}-\rho_{S}\right]}^{2}$ can be changed over a wide range. The goal is to find the proper solvent conditions from which the matching condition, namely $d\Sigma /d\Omega \left(Q\right)\propto {\Delta \rho }^{2}=0$ at ρ_P_ = ρ_S_ (Equations (A1) and (A2)), can be determined, i.e., the scattering length density ρ_P_ of the polymer. For the identification of the polymer, the experimental ρ_P_ has to be compared with the calculated ρ in Table 2.

Well-known and often used contrast mediums are mixtures of H_2_O and D_2_O at different ratios. Figure 1A shows the average coherent scattering length density (ρ_S_) of water molecules versus D_2_O concentration in H_2_O. It is evident that there is a continuous change of ρ_S_ from −0.561 to 6.372 10^10^ cm^−2^. The isotope effect on scattering is explained by the interaction of neutrons with the nucleus of atoms thereby delivering individual values for the coherent scattering length for isotopes of the same element as tabulated in [15]. The scattering length densities of the polymers in Table 2 are determined from the atomic coherent scattering length and monomer molar volume according to $\rho =\sum_{j}b_{j}{/\Omega }_{M}$.

SCFs are less known as contrast media in SANS, i.e., fluids at temperatures and pressures above the critical point. These media have the important advantage of only needing one membrane and ρ_S_ can be sensitively tuned by pressure. This is in contrast to H_2_O/D_2_O, in which one needs a new membrane for each mixture, which is much more time consuming and prone to errors.

In this study, we used carbon dioxide (CO_2_) and deuterated methane (CD_4_) as contrast media. Figure A2a,b show the isotherms of ρ_S_ for CO_2_ at 10, 23.8 and 38 °C and CD_4_ for 10 °C for pressures between 1 and 500 bar. A recent SANS study, with CO_2_ as SCF at 45 °C, shows the effect of pressure on the thermal number density fluctuations [48]. In this reference, we also describe the equipment of the pressure cell used for this experiment. For CO_2_ at 10 °C and 23.8 °C a first order gas/liquid phase transition is seen from the jump of ρ at P = 62.5 bar and 45.1 bar, respectively, whereas the other two isotherms show ρ_S_ beyond the critical point, i.e., in the SCF-regime beyond 31 °C/73.8 bar and −82.6 °C/45.9 bar for CO_2_ and CH_4_, respectively. This indicates a transition of the gases from low pressure to SCF at higher pressure, thereby enabling a gradual increase of ρ_S_ by varying the pressure where CO_2_ and CD_4_ cover a large range of values for ρ_S_ up to 500 bar. On the other hand, CH_4_ cannot work as contrast medium because its ρ_S_ is negative due to the negative coherent scattering length of the hydrogen (b_H_ = −3.739×10^−13^ cm) [15].

Ideally, the scattering of homogenous polymeric materials should become zero at the matching condition when the contrast medium is filling all membrane pores. However, such condition is seldom fulfilled in RO membranes, thereby showing finite scattering at the minima of the matching scattering length density, ρ_m_. Furthermore, R_g_ changes with ρ_S_ showing as for the SW30HR and BW30LE membranes a maximum at the matching condition (ρ_m_) (Figure 5b and Figure 6b). The expression of the experimental R_g_ in Equation (A9) is described as a function of ρ_S_ [37],

(A9) $$R_{g}^{2}\left(\rho_{S}\right)=\left[I_{\mathrm{cl}}\left(0\right)\times R_{g,\mathrm{cl}}^{2}\times \rho_{P}^{2}+I_{\mathrm{op}}\left(0\right)\times R_{g,\mathrm{op}}^{2}\times {\left(\rho_{P}-\rho_{S}\right)}^{2}\right]/\left[I_{\mathrm{cl}}\left(0\right)\times \rho_{P}^{2}+I_{\mathrm{op}}\left(0\right)\times {\left(\rho_{P}-\rho_{S}\right)}^{2}\right]$$

$I_{A}\left(0\right)={d\Sigma /d\Omega }_{A}\left(0\right)/{\left(\rho_{A}-\rho_{S}\right)}^{2}$ as well as the R_g_’s of the open and closed pores. Equation (A9) is derived from the sum of scattering of the two pore contributions in Equation (A10), both

(A10) $${d\Sigma /d\Omega }_{A}\left(Q\right)≅{d\Sigma /d\Omega }_{A}\left(0\right)\left[1+R_{A}^{2}Q^{2}/3\right]\mathrm{and}\;{d\Sigma /d\Omega }_{A}\left(Q\right)={d\Sigma /d\Omega }_{\mathrm{op}}\left(Q\right)+{d\Sigma /d\Omega }_{\mathrm{cl}}\left(Q\right)$$

approximated by the first two terms of the Taylor expansion of Guinier’s law [26] (p167)). In vacuum, i.e., ρ_S_ = 0 one gets: with $\gamma =I_{\mathrm{cl}}(0)/\left[I_{\mathrm{cl}}{(0)+I}_{\mathrm{op}}\left(0\right)\right]$. The subscripts “op” (open) stands for pores accessible for fluids in homogenous polymeric material, and therefore: ${d\Sigma /d\Omega }_{\mathrm{op}}\propto {\left(\rho_{P}-\rho_{S}\right)}^{2}$, whereas “cl” (closed) arises from scattering of an internal inhomogeneity whose R_g_ is independent of ρ_S_, i.e., ${d\Sigma /d\Omega }_{\mathrm{cl}}\left(0\right)\propto {\left(\rho_{P1}-\rho_{P2}\right)}^{2}$. For closed pores ${d\Sigma /d\Omega }_{\mathrm{cl}}\left(0\right)\propto \rho_{P}{}^{2}$. The two radii R_g_,_op_ and R_g,cl_ are determined from R_g_ at the contrasts ρ_S_ = 0 and ρ_S_ = ρ_P_, i.e., for vacuum and matching condition, respectively.

### Appendix A4. PALS in Polymers

In polymers, the lifetime of ortho-Positronium (o-Ps), a bound state of positron and electron, is correlated with the void size where o-Ps annihilates the electrons at the void wall by interaction (pick-off process). The so-called Tao–Eldrup model (e.g., ref. [49] (Appendix)) describes the relation between the lifetime (τ) of o-Ps, which is assumed to be in its ground state, and the void size described by a sphere with radius R_0_. A smaller radius R is defined as a kind of hard sphere radius, whereby ΔR = R_0_ – R describes the distance of the outer shell with non-zero electron density. In this model the relation between lifetime τ and radii is given by

(A11) $$\tau \left[\mathrm{ns}\right]=0.5\times {\left[1-\frac{R}{R_{0}}+\frac{\sin}{2\pi }\right]}^{-1}$$

the empiric parameter ΔR = R_0_ − R was shown to be 1.656 Å [20].

Depth resolved determination of the pore size becomes possible by a pulsed positron beam with variable positron energy [22,23]. A positron flux of 10^9^ s^−1^ at a kinetic energy of 1 keV provided by the positron source NEPOMUC at MLZ enables such a pulsed low-energy positron system (PLEPS) [21,22]. The mean implantation depth $\bar{z}$ for a given positron energy E can be approximated by the relationship

(A12) $$\bar{z}=\left({A/d}_{M}\right)E^{n}$$

where the material mass density d_M_ and the material dependent parameters A and n, which can be found in literature (e.g., [20]). For the present PALS calculations, we use the values of A = 2.8 µg·cm^−2^·keV^−n^ and n = 1.7, respectively.

Within polymeric systems, positrons annihilate according to at least three decay channels with different decay times τ_i_ (i = 1,2,3,..). The lifetimes τ_1_ and τ_2_ are related to direct positron annihilation (without positronium formation) whereby the shortest lifetime τ_1_ also comprises the annihilation of para-positronium (p-Ps). Both are in a 100 ps time range. The longer component τ_3_ is associated with the so-called pick-off lifetime of o-Ps in matter and extends to some ns. The time resolution of the instrument is in the range of 250 ps [22]. For data evaluation, the spectra are numerically deconvoluted by the measured resolution function and fitted with a least-squares method based on the Levenberg–Marquardt algorithm. For several spectra, reasonable fits are only obtained by taking into account an additional longer lifetime component with τ_4_ > τ_3._ In this time regime the Tao–Eldrup model is not valid anymore, but pore sizes can be estimated using a classical description of the positronium–wall interaction [35].Figure A3 shows a typical positron lifetime spectrum exhibiting four lifetimes τ_i_, (i = 1–4). The decomposition into four components results in a reliable agreement between fit and recorded data as confirmed by the residuum (below). Note, that spectra recorded at PLEPS show a very low constant background of 1.3 counts per channel only. The intensity I_3_ allows us to observe changes in the free volume, as it is directly related to the total free volume of polymer melts. However, a reasonable value of free volume fraction on an absolute scale cannot be given, although I_3_ is considered to be proportional to the number of pores with a mean radius determined by τ_3_.s

## Abbreviations

SANS	small-angle neutron scattering
PALS	positron-annihilation lifetime spectroscopy
dΣ/dΩ(Q)	differential macroscopic cross-section
dΣ/dΩ_inc_	incoherent scattering cross-section_._
δ	scattering angle
λ	wavelength of neutron
k	wavenumber of neutron (2π/λ)
Q	momentum transfer defined as Q=4π/λsin(δ/2)
Q2	second moment with meaning of the invariant of scattering,
b_i_	coherent scattering length of atom “i”
Ω_M_	volume of molecule “M”
ρ_M_	coherent scattering length density of molecule “M” (ρ_M_ = Σ b_i_ / Ω_M_ )
Φ_match_	concentration of D_2_O in mixture of H_2_O/D_2_O showing the same ρ of the sample
R_g_	radius of gyration
P_4_	Porod constant
α	exponent of power law of dΣ/dΩ at Q > 1/R_g_
V_p_, S_p_, N_p_, Φ_p_	volume, surface, number density, and volume fraction of domains exposed to scattering.
D_S_	thickness of sample
RO	reverse osmosis
NF	nanofiltration
TFC	thin film composite
TMP	trans membrane pressure
SSE	simulated secondary effluent
SCF	supercritical fluid
MA	methacrylic acid
